# BIG3 and BIG5 Redundantly Mediate Vesicle Trafficking in *Arabidopsis*

**DOI:** 10.3390/biom11050732

**Published:** 2021-05-14

**Authors:** Yiping Suo, Fenhong Hu, Haojie Zhu, Di Li, Rui Qi, Jirong Huang, Wenjuan Wu

**Affiliations:** Shanghai Key Laboratory of Plant Molecular Sciences, College of Life Sciences, Shanghai Normal University, Shanghai 200234, China; yipingsuo@163.com (Y.S.); fenhonghu@shnu.edu.cn (F.H.); zhuhaojiedna@163.com (H.Z.); 15216620035@163.com (D.L.); qirui_77@163.com (R.Q.); huangjr@shnu.edu.cn (J.H.)

**Keywords:** ARF-GEF, BIG, vesicle trafficking, PIN2, cell proliferation, *Arabidopsis*

## Abstract

Vesicle trafficking plays an important role in delivering a diverse range of cargoes between different membranous systems in eukaryotes. It is well documented that the brefeldin A (BFA)-inhibited guanine nucleotide exchange factor (GEF), named BIG, regulates vesicle budding at the *trans-*Golgi network (TGN) and recycling endosomes through activating the ADP-ribosylation factor (ARFs). Among the five BIGs in *Arabidopsis*, BIG5 is characterized to mediate ARF-dependent trafficking at the plasma membrane or endosomes while the members from BIG1 to BIG4 (BIG1-BIG4) at the TGN in the secretory pathway. However, evidence is increasing to suggest that BIG5 can function redundantly with BIG1-BIG4 to regulate vesicular trafficking in response to various intra- and extra-cellular stimuli. In this study, our genetic analysis showed that BIG5 played an overlapping role at least with BIG3 in cell proliferation. To elucidate molecular mechanisms underlying the BIG5- and BIG3-regulated biological processes, we examined the effect of BIGs on expression patterns of the two transmembrane proteins, PINFORMED 2 (PIN2) epically localized in root epidermal cells and the regulator of G protein signaling 1 (RGS1) localized in the plasma membrane. Our data showed that the PIN2 polar distribution was slightly reduced in *big3 big5* in the absence of BFA, and it was significantly reduced by the treatment of 0.1 µM BFA in *big3 big5*. Further analysis revealed that BFA bodies derived from the plasma membrane were only observed in wild type (WT), *big3* and *big5* cells, but not in the *big3 big5* cells. These results indicate that BIG5 and BIG3 are functionally redundant in the endosome recycling pathway from the plasma membrane to TGN. On the other hand, the single *BIG3* or *BIG5* mutation had no effect on the plasma membrane expression of RGS1, whereas the double mutations in *BIG3* and *BIG5* led to a significant amount of RGS1 retained in the vesicle, indicating that BIG3 and BIG5 act redundantly in mediating protein trafficking. Furthermore, transmission electron microscopy assays showed that Golgi ultrastructure in *big3 big5* cells was abnormal and similar to that in BFA-treated WT cells. Taken together, our data provide several new lines of evidence supporting that BIGs play a redundant role in vesicular trafficking and probably also in maintaining the Golgi structural integrity in *Arabidopsis*.

## 1. Introduction

In eukaryotes, vesicle trafficking is a fundamental cellular process that delivers diverse cargoes between organelles of the secretory and endocytic pathways. This endomembrane traffic system is also essential to preserve organelle structural integrity and specific composition [1,2]. Generally, the transport initiates from vesicle budding from a donor compartment, and subsequently targets to and fuses with an acceptor compartment. It has been well documented that small guanine nucleotide binding proteins (G proteins) act as a molecular switch that can hydrolyze GTP as a common mechanism to regulate vesicle formation and fusion. Small G proteins, which have been classified into five groups including Ras (Rat sarcoma), Rho (Ras homology), Rab (Ras-like in brain), Ran (Ras-like nuclear) and Arf/Sar (ADP-ribosylation factor/secretion-associated and *ras*-superfamily-related gene) [3], are activated by membrane-recruited guanine nucleotide exchange factors (GEFs) through substituting of GDP in the inactive G protein state for GTP. Activated GTP-bound G proteins bind to the membrane and interact with their downstream effectors, such as coat proteins (COPI and clathrin) that further recruit cargoes, and other factors required for cargo sorting and vesicle formation and scission. The active small G proteins are inactivated by GTPase accelerating proteins (GAPs), and then released from the coat complex. Thus, elucidating regulatory mechanisms of GTPase activity is a key step toward our understanding of the complex network for intracellular vesicular trafficking pathways.

To date, the small G protein system composed of ARF and its GEF proteins has been demonstrated to play a critical role in vesicle budding of the secretory pathway [1]. In contrast to other small G proteins that contain lipid modification motifs at the C-terminus, ARF GTPases are recruited to membrane through myristoylation of the N-terminus [4]. Based on their structure and function, the six mammalian ARFs are grouped into three classes: class I (ARF1-3), class II (ARF4-5) and class III (ARF6) [5]. Interestingly, *Arabidopsis* encodes 21 ARF members, including 3 SAR1/ARF1 GTPases that contain no N-myristoylated site, 12 ARF GTPases that are highly similar to the mammalian class I ARF GTPases and 6 ARF-like proteins [4,6]. The ARF GTPases are further divided into four distinct clades, named ARFA to ARFD [4]. It has been demonstrated that ARFA1 members are involved in various intracellular trafficking processes, such as vesicle secretion from the ER to the Golgi, endocytosis and recycling [7]. The ARF GTPases are activated by ARF-GEF proteins, which are characterized by the highly conserved catalytic SEC7 domain facilitating ARF binding and exchange of GDP with GTP, and are divided into three subfamilies, namely small (smaller than 80 kD), medium (from 100 to 150 kD) and large (larger than 170 kD). The ARF-GEF activity of the catalytic SEC7 domain can be blocked by the fungal toxin brefeldin A (BFA), leading to the formation of BFA-induced aggregated bodies [8].

The *Arabidopsis* genome encodes only the large ARF-GEF subfamily members, which fall into the Gea1/2p-like and Sec7p/BIG-like clades [9]. The former subfamily comprises GNOM, GNOM-LIKE1 (GNL1) and GNL2, while the later one comprises five members (BIG1, BIG2, BIG3, BIG4 and BIG5). It has been reported that GNOM and GNL1 are localized and activate ARF1 at the Golgi to mediate retrograde traffic to COPI vesicles from the Golgi stack to the ER [10,11]. GNL2 is specifically expressed in pollen, but can functionally substitute for both GENOM and GNL1 when ectopically expressed [12]. In addition, GNOM is required for maintaining the proper TGN structure and is indirectly involved in protein recycling [13,14]. With regard to functions of the five BIGs, BIG5 mediates endocytosis from the plasma membrane to the TGN and pathogen response, whereas BIG1-BIG4 are functionally redundant in vesicle trafficking from the TGN to the plasma membrane, vacuole and cell division plane [10,15,16]. Besides ARF1, both BIG5 and BIG1-BIG4 can activate ARFA and possibly ARFB, which is not essential for endomembrane trafficking pathways [17]. Recently, Xue et al. (2019) [18] reported that the *big3 big5* double mutant displayed more severe phenotypes in leaf and root growth, and root gravitropism than the *big5* single mutant, indicating of their overlapping roles in plant growth and development. Further analysis showed that BIG5 and BIG3 participated in brassinosteroid (BR) signaling through regulating BR receptor1 (BR1) trafficking. In this study, we analyzed overlapping functions between BIG3 and BIG5 in plant growth and development, and found that BIG3 and BIG5 are functionally redundant in vesicle trafficking.

## 2. Materials and Methods

### 2.1. Plant Materials and Growth Conditions

The *big3* (SALK_044617) and *big5* (SALK_013761) are obtained from the ABRC stock center (https://abrc.osu.edu/, accessed on 14 May 2021). All these seeds were in the wild type (WT) Col-0 background. The *big3 big5* double mutant was isolated from the F_2_ population of the cross between *big3* and *big5*. The transgenic plants expressing *pPIN2::PIN2-GFP* were crossed to *big3 big5*, and the plants with *pPIN2::PIN2-GFP* in *big3 big5* were isolated from the F_2_ population. The surface-sterilized seeds were stratified at 4 °C in the dark for 2 to 3 days, and then germinated on the half-strength Murashige and Skoog (MS) medium (pH 5.7) (Sigma-Aldrich, St. Louis, MO, USA) containing 0.7% agar and 1% sucrose at 22 °C with a light intensity of 100 µmol m^−2^ sec^−1^ in long day conditions (16 h light/8 h dark). The 7-day-old seedlings were transplanted to soil and grown under the same condition as above.

### 2.2. Phenotypic Analysis

For phenotypic characterization of cotyledons, 7-day-old WT, *big3*, *big5* and *big3 big5* plants were photographed, and cotyledon length was measured using image J software and statistically analyzed. Meanwhile, seedlings were treated with 10 μM FM4-64 for 30 min and cotyledons were observed under the fluorescent microscopy Olympus 3000. The number of pavement cells every 100 µm^2^ were counted and the cell number in each cotyledon was calculated. For phenotypic characterization of rosette leaves, the length of the third true leaf from 20-day-old wild type, *big3*, *big5* and *big3 big5* plants were measured.

Root length of 7-day-old WT, *big3*, *big5* and *big3 big5* seedlings cultured vertically were measured directly, or stained with 10 μg/mL PI for 3 min before observation under the microscopy Olympus 3000. The root meristem size was measured from the quiescent center to the end of the meristem and meristem cell number was counted correspondingly.

### 2.3. Plasmid Construction and Transformation

The RGS coding DNA sequence (CDS) was cloned into the pENTR SD/D-TOPO entry vector (Invitrogen, Carlsbad, CA, USA), recombined into the p2GWY7 vector (https://gatewayvectors.vib.be/collection/p2gwy7, accessed on 14 May 2021) and transformed into the *Arabidopsis* protoplasts according to the method described by Wu et al. (2009) [19]. The YFP signal was observed using the Olympus 3000 according to the protocol guide.

### 2.4. BFA Sensitivity, PIN2 Polarity and FM4-64 Uptake Analysis

To test BFA sensitivity of WT, *big3*, *big5* and *big3 big5*, 2-day-old seedlings cultured on the 1/2 MS medium were transplanted to the 1/2 MS containing 0.1 µM, 0.25 µM, 0.5 µM, 1 µM and 2.5 µM BFA (DMSO as a control) for another 5 days. The root length was measured and statistically analyzed using Image J.

Transgenic seeds with *pPIN2::PIN2-GFP* in wild type and in *big3 big5* were germinated on the 1/2 MS for 2 days, and then were transferred to the 1/2 MS containing DMSO and 0.1 µM BFA for another 5 days. GFP signals on the cortical cells and epidermal cells were observed under the Olympus 3000. The integrated fluorescence densities of lateral and polar areas of each cell were measured using Image J, and the lateral: polar ratio was then calculated and statistically analyzed.

For characterization of FM4-64 uptake, 7-d-old WT, *big3, big5* and *big3 big5* seedlings were treated with 10 μM FM4-64 for 10, 30 and 120 min, or with 10 μM FM4-64 and 25 μM BFA simultaneously for 30 min. Fluorescence of FM4-64 was observed under the Olympus 3000.

### 2.5. Analysis of Subcellular Localization and Golgi Ultra-Structure

Analysis of the Golgi subcellular localization was carried out by fusing the fluorescent protein with the cytoplasmic tail and the transmembrane domain (first 49 aa) of GmMan1, the soybean α-1,2-mannosidase I [20]. The transgene *Golgi-RFP* was transferred into the *big3 big5* mutant background by crossing transgenic plants expressing Golgi-RFP with *big3 big5*. To observe the effect of the mutation of *BIG3* and *BIG5* on the Golgi structure, two-day-old seedlings germinated on the 1/2 MS were transferred to the 1/2 MS containing DMSO or 0.1 µM BFA. Golgi-RFP in cortical cells of the meristematic area and in epidermal cells of the elongation zone was observed with confocal laser scanning microscope (Olympus 3000, Olympus, Tokyo, Japan).

For Golgi ultra-structure observation, WT and *big3 big5* leaves cultured on 1/2 MS medium with or without BFA treatment were cut into small pieces and fixed in a solution of 4% glutaraldehyde, and then processed, embedded and viewed under a transmission electron microscopy.

## 3. Results

### 3.1. BIG3 and BIG5 Function Redundantly to Regulate Plant Growth via Cell Proliferation

**Figure 1:**
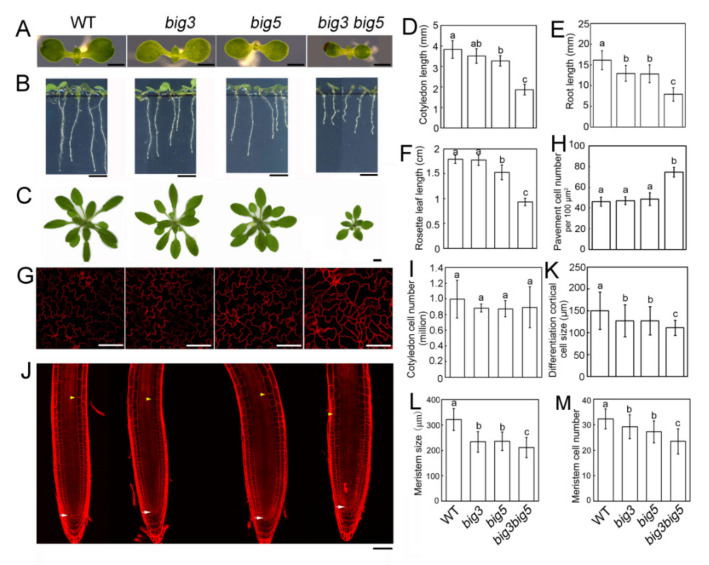
Phenotypic analyses of WT, *big3*, *big5* and *big3 big5* plants. (**A**) cotyledon phenotypes of 7-d-old WT, *big3*, *big5* and *big3 big5* seedlings. Bars = 1 mm. (**B**) root phenotypes of 7-d-old WT, *big3*, *big5* and *big3 big5* seedlings. Bars = 3 mm. (**C**) representative phenotypes of 20-d-old WT, *big3*, *big5* and *big3 big5* plants. Bars = 1 cm. (**D**) quantification of the cotyledon length in (**A**). (**E**) quantification of the root length in (**B**). (**F**) quantification of the third pair of rosette leaf length in (**C**). (**G**) confocal microscopy images of cotyledon pavement cells shown in (**A**). The cotyledons were stained with 10 μM FM4-64 for 30 min before observation. (**H**,**I**) quantification of the pavement cell number in (**G**). (**J**) root meristem phenotypes of 7-d-old WT, *big3*, *big5* and *big3 big5* seedlings. The seedlings were stained with 10 μg/mL PI for 5 min. Bars = 50 μm. White arrowheads indicate the quiescent center and yellow arrowheads indicate the end of the meristem. (**K**–**M**) quantification of the differentiation cortical cell size (**K**), the root meristem size (**L**) and meristem cell number (**M**). All the values are presented as mean ± sd (*n* > 10), and analyzed with one-way ANOVA and multiple comparisons (*p* < 0.05, different letters denote statistically significant differences).

We previously reported that BIG1 and BIG5 are essential for male gametophyte transmission in *Arabidopsis* [21]. We also observed functional redundancy between BIG3 and BIG5 in plant growth and development (Figure 1). Except for a shorter root, the *big3* single mutant had the same size as the wild type (WT) with regard to cotyledons and rosette leaves (Figure 1A–F). However, the *big5* single mutant was significantly shorter in cotyledon length, root length and rosette leaf length compared to WT (Figure 1A–F). Interestingly, the *big3 big5* seedlings displayed much smaller size than the single mutants (*big3* and *big5*) and WT. The length of the *big3 big5* cotyledon, root and rosette leaf was 48.9%, 48.7% and 52.1% of the WT’s, respectively. These results suggest that knocking out *BIG3* aggravates the dwarf phenotype of *big5*.

We then quantified the cell number and size in cotyledons and root meristems from WT, *big3*, *big5* and *big3 big5* seedlings. Our results showed that the pavement cell number per unit area had no significant difference between *big 3* or *big5* and WT, but was significantly higher in *big3 big5* than in WT and two single mutants (Figure 1H). In contrast, the total pavement cells per cotyledon in *big3 big5* had no significant differences compared to WT and two single mutants (Figure 1I). In addition, we found that the lobes of *big3 big5* pavement cells were not well formed (Figure 1G). These results indicate that expansion and/or development of pavement cells are severely inhibited in *big3 big5*. With regard to root phenotypes, *big3 big5* exhibited shorter cell length in the mature zone and shorter root meristem than the two single mutants, which were further shorter than WT (Figure 1J–M). Taken together, our data suggest that BIG3 and BIG5 function redundantly in cell proliferation.

### 3.2. Root Growth of big3 big5 Is Hypersensitive to BFA

**Figure 2:**
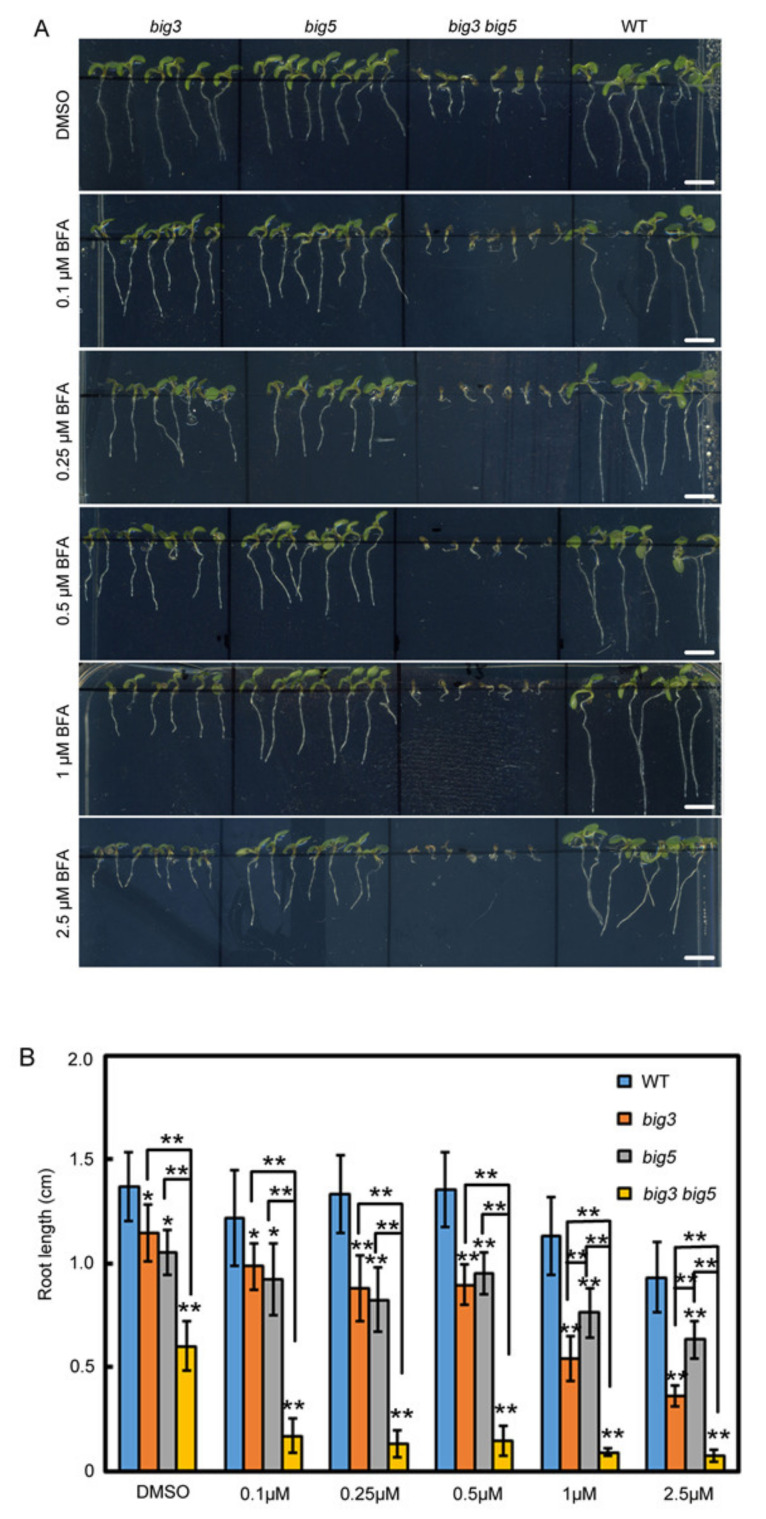
Root sensitivity of WT, *big3*, *big5* and *big3 big5* seedlings to BFA. (**A**) two-day-old WT, *big3*, *big5* and *big3 big5* seedlings germinated on 1/2 MS medium were transferred to the medium containing different concentrations of BFA (DMSO as a control). Bars = 0.5 cm. (**B**) quantification of the root length in (**A**). The values are presented as mean ± sd (n = 15), and analyzed with one-way ANOVA and multiple comparisons (*, *p* < 0.05; **, *p* < 0.01).

To pharmacologically confirm the functional redundancy between BIG3 and BIG5, we analyzed effects of *BIG* mutations on the sensitivity to the fungal toxin Brefeldin A (BFA), an inhibitor of ARF-GEFs. To accomplish this, seeds were germinated on 1/2 MS medium for two days, and then the seedlings were transferred to the medium containing DMSO (control) or five different BFA concentrations as indicated in Figure 2. Without BFA treatment, *big3* *big5* had the shortest roots among the tested genotypes, and the second and third shorter roots were *big5* and *big3*, respectively, compared to WT. Interestingly, the *big3* mutant produced shorter roots than *big5* at the BFA concentrations higher than 1.0 μM (Figure 2B), indicating that root growth of *big3* was more sensitive to BFA than that of *big5*. Consistently, the *big3 big5* double mutant showed an extremely BFA sensitive phenotype in root growth. The root growth of *big3 big5* was severely inhibited even as low as 0.1 μM of BFA, at which the root length of *big3* and *big5* single mutants was 80.9% and 75.8% of WT. Therefore, our results indicate that both BIG3 and BIG5 regulate root growth in a BFA-dose dependent manner, although BIG3 plays a key role in resistance to BFA.

### 3.3. PIN2 Polarity in big3 big5 Roots Is Sensitive to BFA

**Figure 3:**
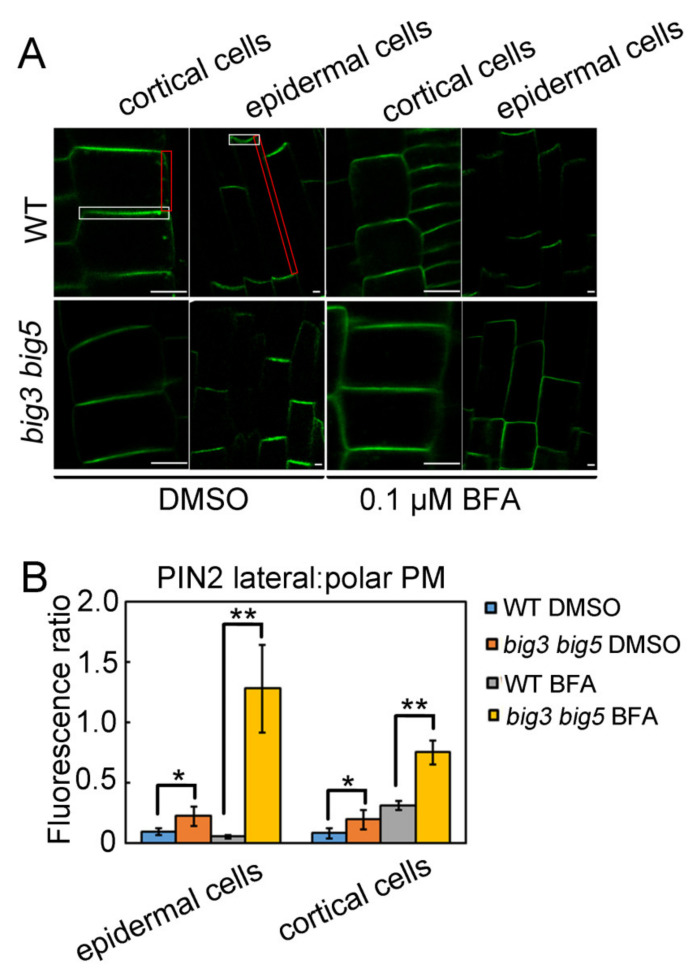
PIN2 polarity of *big3 big5* roots is sensitive to BFA. (**A**) seedlings germinated on 1/2 MS medium for 2 days were transferred to the medium containing 5 µM DMSO (control) or 0.1 µM BFA for additional 5 days. The GFP signals were analyzed with confocal microscope. Bars = 10 µm. (**B**) quantification of lateral to polar plasma membrane signal as shown from (**A**). Red rectangles indicate lateral plasma membrane and white rectangles indicate lateral plasma membrane. The significant difference was calculated by a two-tailed Student’s *t* test, *, *p* < 0.05, **, *p* < 0.001. Images are representative from three repeats.

Considering that auxin plays a key role in root growth, we examined whether the sensitivity of *big3 big5* roots to BFA is associated with the PIN polar subcellular localization. Thus, we made the *big3 big5* double mutant containing the *pPIN2::PIN2-GFP* construct by crossing the *pPIN2::PIN2-GFP* transgenic line with *big3 big5*. As previously reported, in WT roots, PIN2 exhibits apical localization in root epidermal cells and basal localization in cortical cells without BFA treatment (Figure 3A). Although almost the same polar distribution of PIN2 was observed in *big3 big5* in the absence of BFA (Figure 3A), quantification analysis of GFP fluorescence showed that the ratio of lateral to polar plasma membrane fluorescence intensity was higher in *big3 big5* than in WT both in cortical cells and epidermal cells (Figure 3B). The treatment of 0.1 µM BFA did not alter PIN2 polarity in WT cortical and epidermal cells, but dramatically changed the polar localization of PIN2, which was almost evenly distributed in the whole plasma membrane in *big3 big5* cortical and epidermal cells (Figure 3). These results indicate that BIG3 and BIG5 are functionally redundant in regulating PIN2 polar localization in roots.

**Figure 4:**
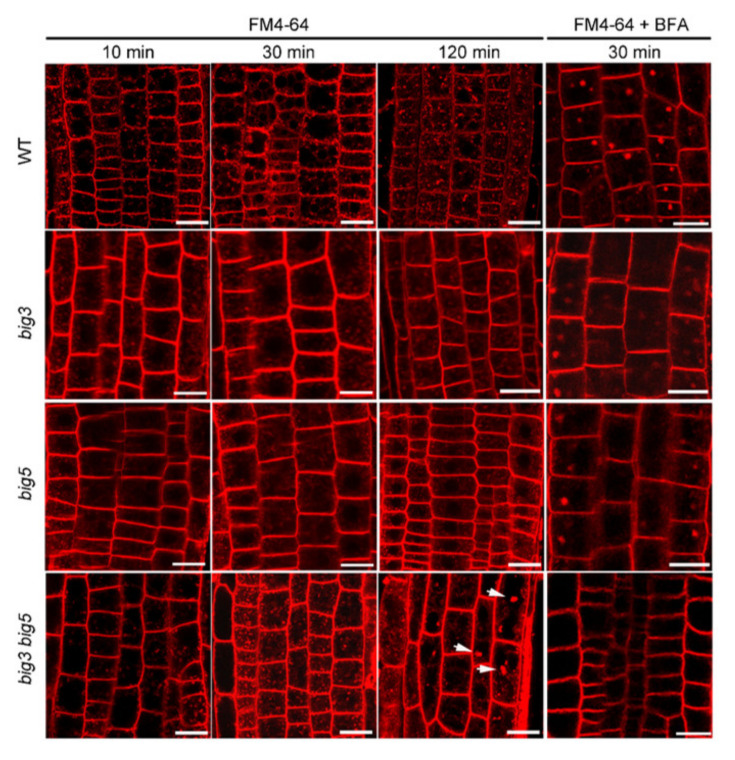
BIG3 and BIG5 are functionally redundant in vesicle formation from the plasm membrane. Confocal microscopy analysis of FM4-64 uptake and vesicle trafficking in roots of 7-d-old WT, *big3*, *big5* and *big3 big5*. Seedlings were treated with 10 μM FM4-64 for 10, 30 and 120 min, or with 10 μM FM4-64 and 25 μM BFA simultaneously for 30 min. Arrowhead indicate the bigger vesicles such as BFA bodies in *big3 big5* cells 120 min after FM4-64 treatment. Bars = 15 µm.

Since endocytosis is crucial for PIN2 polar localization, we proposed that BIG3 and BIG5 mutations might affect the vesicle trafficking pathway. To testify this hypothesis, we investigated the difference in endocytosis between WT and *big3 big5* by treating roots with FM4-64, an amphiphilic styryl molecular marker for endocytosis. Once a cell uptakes FM4-64, the plasma membrane (PM), *trans-*Golgi network (TGN), multivesicular bodies (MVB) and vacuole will be subsequently stained step by step through endocytosis. As shown in Figure 4, red and small vesicles were detected in WT, *big3*, *big5* and *big3 big5* cells 10 min after FM4-64 treatment. The size of intracellular vesicles was kept in a constantly stable state in WT, *big3* and *big5* cells during 2 h observation. In contrast, bigger vesicles such as BFA bodies were detected in *big3 big5* cells 120 min after FM4-64 treatment (Figure 4). These results suggest that BIG3 and BIG5 are involved in the endocytotic pathway. To confirm this result, we treated seedlings simultaneously with FM4-64 and BFA. FM4-64-contained BFA bodies were clearly detected in WT, *big3* and *big5* cells, but absent in *big3 big5* cells 30 min after BFA treatment, indicating that the endocytotic pathway in root endothelial cells of *big3 big5* was altered, probably due to a slower rate of vesicle trafficking. Taken together, our data indicate that BIG3 and BIG5 are functionally redundant in the endocytotic pathway from the PM to TGN.

### 3.4. Golgi Ultrastructure Is Altered in big3 big5

**Figure 5:**
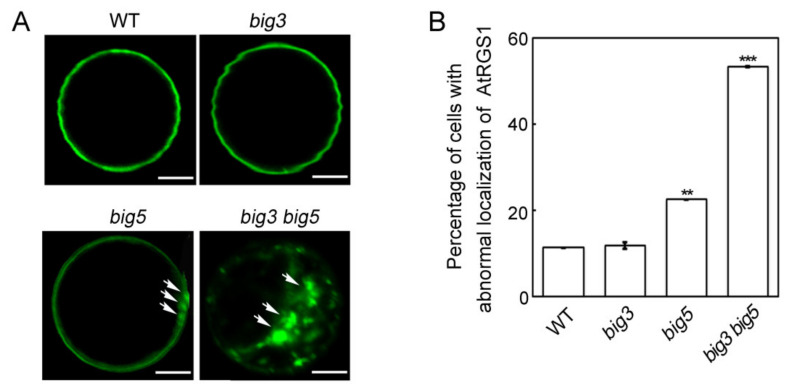
Subcellular localization of AtRGS1 in WT, *big3*, *big5* and *big3 big5* protoplasts. (**A**) the fusion protein AtRGS1-YFP was transiently expressed in protoplasts from WT, *big3*, *big5* and *big3 big5* cultured at 1/2 MS for 10 days. YFP fluorescence was observed 17 h later. Typical protoplasts of each genotype were shown. Bars = 10 μm. White arrow heads indicate the dots in the cytoplasm in *big5* and *big3 big5* protoplasts. (**B**) percentage of cells with GFP signals retained in the cytoplasm. The values are presented as mean ± sd (n = 50). Asterisks indicate significant differences, compared to WT (**, *p* < 0.01; ***, *p* < 0.001. Student’s *t* test). Three independent experiments were performed.

**Figure 6:**
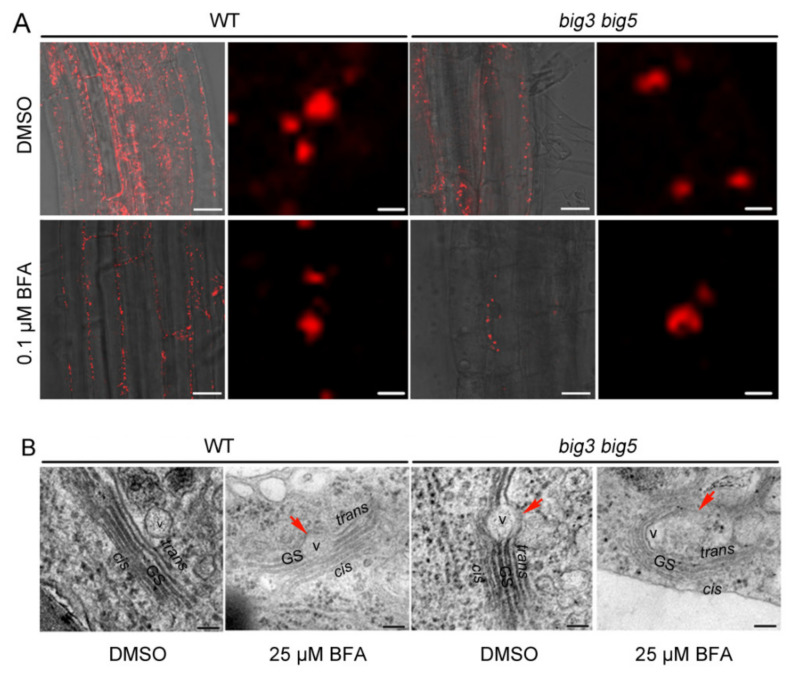
The Golgi structure in WT and *big3 big5* with or without BFA treatment. (**A**) the WT and *big3 big5* seeds expressing Golgi-RFP were germinated on 1/2 MS medium for 3 days, and then the seedlings were transferred to 1/2 MS medium containing 1/1000 DMSO and 0.1 μM BFA for 5 days. The morphology of Golgi apparatus was observed under confocal microscopy. The right lane (Bars = 2 μm) of each genotype is the enlarged image of the left lane (Bars = 20 μm). (**B**) the ultrastructure of Golgi in WT and *big3 big5* cultured on 1/2 MS or treated with 25 μM BFA for 4 days. Bars = 100 nm. GS, Golgi stacks; V, vesicle; arrow heads, abnormal vesicle-like structure.

To examine whether Golgi ultrastructure is affected in the *big3 big5* double mutant, we first transiently expressed GFP-fused AtRGS1, which is a regulator of G protein signaling and localized in the plasma membrane, in WT, *big3*, *big5* and *big3 big5* protoplasts. Our results showed that GFP signals were localized predominantly in the plasma membrane of the WT and *big3* protoplasts, whereas there were about 30% and 60% of the cells with GFP signals retained in the cytoplasmic dots in *big5* and *big3 big5* protoplasts, respectively (Figure 5). These results suggest that BIG3 and BIG5 may be involved in regulating the transport of AtRGS1 from the Golgi apparatus to the plasma membrane. To confirm this hypothesis, we transformed a Golgi marker (Golgi-RFP) in the WT and *big3 big5* genetic backgrounds. Two-day-old *Golgi-RFP/WT* and *Golgi-RFP/big3 big5* seedlings germinated on 1/2 MS medium were transferred to the 1/2 MS medium containing with or without 0.1 μM BFA for additional 5-day vertical growth. Confocal microscopy analysis showed that Golgi-RFP signals exhibited round solid dots in WT and *big3 big5* in root cells without BFA treatment; 0.1 μM BFA treatments had no effect on the shape of the RFP-labelled Golgi in WT cells, but led to generation of the concave Golgi shape in *big3 big5* cells (Figure 6A). Then, transmission electron microscopy was used to examine the Golgi ultrastructure in WT and *big3 big5* mutants. We found that WT cells had a typical ultrastructure of the Golgi and the *trans-*Golgi network under normal growth conditions, whereas 25 μM BFA treatments apparently inhibited vesicle secretion from the *trans*-side of the Golgi apparatus (Figure 6B, left), because a larger vesicle was formed in the *trans*-Golgi apparatus. However, abnormal Golgi ultrastructure, which was similar to that in BFA-treated WT cells, was observed even in BFA-untreated *big3 big5* cells, and severely altered ultrastructure of the Golgi, which encircled larger vesicles, was detected in BFA-treated *big3 big5* cells (Figure 6B, right). These results suggest that BIG3 and BIG5 are involved in the formation of vesicles on the *trans*-side of Golgi stacks.

## 4. Discussion

It has been generally recognized that plant BIGs regulate post-Golgi membrane trafficking. However, the roles of different BIGs in protein secretion, endocytosis and exocytosis remain largely unknown. In this study, we showed that the *big3 big5* plant was significantly smaller than the single *big5* and *big3* mutants (Figure 1), and knocking out *BIG3* aggravates the dwarf phenotype of *big5*, suggesting that BIG5 and BIG3 had the overlapping roles in plant growth and development (Figure 1). Recently, Xue et al. (2019) [18] also showed that BIG5 functions redundantly with BIG3 in plant growth and gravitropism. We also analyzed other *big* multiple mutants, and found that *big2 big4 big5* was much smaller than *big2 big5* or *big4 big5* in plant body despite that the plant size of *big2 big5* and *big4 big5* was smaller than *big5* (Supplementary Figure S1). We previously reported that BIG1 and BIG5 could act in the same genetic pathway to regulate male gametophyte transmission [21]. Thus, it is possible that all BIGs act in a partially overlapping manner in the intracellular vesicle trafficking system, indicating that BIG5 can mediate late secretion from the TGN to different target membranes while BIG1–BIG4 regulate vesicle recycling between TGN/endosome and the plasma membrane or other intramembrane systems.

To support the above hypothesis, we investigated the effect of BIGs on the change in subcellular localization of two transmembrane proteins, RGS1 and PIN2. Our data showed that the plasma membrane expression pattern of AtRGS1 was severely blocked in the *big3 big5* protoplasts, but not in the *big3* and *big5* single mutants, indicating that BIG5 and BIG3 mediates the secretion pathway or the vesicle recycling pathway from the TGN/endosome to the plasma membrane. *Arabidopsis* PIN2 protein is required for the transport of the phytohormone auxin from the root tip to the root elongation zone, and is distributed at the apical site of epidermal cells of the root meristem [22]. It is well known that endocytosis plays an essential role in the establishment of PIN polarity. Previous publications showed that GNOM is required for polar localization of the auxin-efflux carrier PIN1 from the trans-Golgi network (TGN) or endosomes to the basal plasma membrane [11,14]. In contrast, Naramoto et al. (2014) showed that GNOM predominantly localized in the Golgi but not in the TGN or early endosome (EE) [13], and the observed disturbance of PIN polarity in *gnom* was associated with the indirect role of GNOM in maintaining the TGN/EE structure. Consistently, Tanaka et al. [23] reported that BIG5/BEN1 localized to TGN, which is distinct from that of GNOM subcellular localization. In addition, BIG3 was also reported to co-localize with endocytic tracer FM4-64 labeling TGN after brief uptake, and accumulated together with FM4-64 in BFA-induced post-Golgi membrane vesicle aggregates [24]. Thus, it is likely that BIGs, instead of GNOM, play a key role in the regulation of the post-Golgi trafficking of membrane proteins including PIN proteins.

Evidence is increasing to indicate that BIG proteins are involved in vesicle trafficking between the TGN to plasma membrane. For example, BIG2/BEN3 is localized in the TGN/EE, and regulates de novo synthesized PIN1 secretion to the plasma membrane [25]. In this study, we found that the PIN2 level decreased at the apical plasma membrane but increased at the lateral plasma membrane in *big3 big5* double mutant, indicating that both BIG3 and BIG5 are required for the PIN2 apical polar localization. Interestingly, no BFA bodies were detected in *big3 big5* if cells were simultaneously treated with FM4-64 and BFA for 30 min (Figure 4). These data suggest that BIG3 and BIG5 are functionally redundant in the process of vesicle trafficking. Furthermore, TEM analysis revealed that the Golgi structure was significantly altered and more sensitive to BFA in *big3 big5*, compared to that in WT (Figure 6), suggesting that blocking vesicle formation at the *trans*-Golgi side or loss-of-function of BIGs might indirectly affect the secretion pathway of membrane proteins from the cis-Golgi to *trans*-Golgi apparatus. In animal cells, the two BIG proteins, BIG1 and BIG2, localize to the two distinct organelles, TGN and endosomes, where they activate ARF1 and ARF3 and play redundant roles in vesicle recycling between the TGN or endosome and the plasma membrane, and between TGN and late endosome [26,27]. Recently, both BIG5 and BIG1-BIG4 can activate ARF1 and ARFA [17], suggesting that BIGs can mediate the same step of the endomembrane trafficking pathway. In the future, dissection of the machinery that controls vesicle trafficking via BIGs is essential for our understanding of the still enigmatic mechanisms, by which distributions of membrane proteins in various cellular compartments are dynamically regulated in plants.

## Data Availability

The data presented in this study are available on request from the corresponding author.

## Supplementary Materials

The following are available online at https://www.mdpi.com/article/10.3390/biom11050732/s1, Figure S1: Phenotypes of WT, *big5*, *big2 big5*, *big4 big5* and *big2 big4 big5*.
